# A comparison of deep learning U-Net architectures for posterior segment OCT retinal layer segmentation

**DOI:** 10.1038/s41598-022-18646-2

**Published:** 2022-09-01

**Authors:** Jason Kugelman, Joseph Allman, Scott A. Read, Stephen J. Vincent, Janelle Tong, Michael Kalloniatis, Fred K. Chen, Michael J. Collins, David Alonso-Caneiro

**Affiliations:** 1 Contact Lens and Visual Optics Laboratory, Centre for Vision and Eye Research, School of Optometry and Vision Science, Queensland University of Technology (QUT), Kelvin Grove, Australia; 2 Centre for Eye Health, University of New South Wales (UNSW), Sydney, NSW Australia; 3 School of Optometry and Vision Science, UNSW, Sydney, NSW Australia; 4 Centre for Ophthalmology and Visual Science (Incorporating Lions Eye Institute), The University of Western Australia, Perth, WA Australia; 5 Department of Ophthalmology, Royal Perth Hospital, Perth, WA Australia; 6 Ophthalmology, Department of Surgery, University of Melbourne, East Melbourne, Victoria Australia

**Keywords:** Computer science, Medical imaging

## Abstract

Deep learning methods have enabled a fast, accurate and automated approach for retinal layer segmentation in posterior segment OCT images. Due to the success of semantic segmentation methods adopting the U-Net, a wide range of variants and improvements have been developed and applied to OCT segmentation. Unfortunately, the relative performance of these methods is difficult to ascertain for OCT retinal layer segmentation due to a lack of comprehensive comparative studies, and a lack of proper matching between networks in previous comparisons, as well as the use of different OCT datasets between studies. In this paper, a detailed and unbiased comparison is performed between eight U-Net architecture variants across four different OCT datasets from a range of different populations, ocular pathologies, acquisition parameters, instruments and segmentation tasks. The U-Net architecture variants evaluated include some which have not been previously explored for OCT segmentation. Using the Dice coefficient to evaluate segmentation performance, minimal differences were noted between most of the tested architectures across the four datasets. Using an extra convolutional layer per pooling block gave a small improvement in segmentation performance for all architectures across all four datasets. This finding highlights the importance of careful architecture comparison (e.g. ensuring networks are matched using an equivalent number of layers) to obtain a true and unbiased performance assessment of fully semantic models. Overall, this study demonstrates that the vanilla U-Net is sufficient for OCT retinal layer segmentation and that state-of-the-art methods and other architectural changes are potentially unnecessary for this particular task, especially given the associated increased complexity and slower speed for the marginal performance gains observed. Given the U-Net model and its variants represent one of the most commonly applied image segmentation methods, the consistent findings across several datasets here are likely to translate to many other OCT datasets and studies. This will provide significant value by saving time and cost in experimentation and model development as well as reduced inference time in practice by selecting simpler models.

## Introduction

Optical coherence tomography (OCT) is a non-invasive, high resolution imaging modality that is commonly used in ophthalmic imaging. By allowing easy visualisation of the retinal tissue, OCT scans can be employed by clinicians and researchers for diagnosis of ocular diseases and monitoring disease progression or response to therapy through the quantitative and qualitative analysis of features within these images. The inner and outer boundaries of the retinal and choroidal layers are of particular interest in OCT image analysis. However, the marking of the position of these boundaries can be slow and inefficient when performed manually by a human expert annotator. Therefore, rapid and reliable automated algorithms are necessary to perform retinal segmentation in a time-efficient manner.

Early automatic methods for OCT retinal and choroidal segmentation relied upon standard image processing techniques as part of methods that were handcrafted to suit particular sets of images^1–9^. While these methods achieve the goal of saving time, the specific nature of the rules that make up their algorithms means that they may not generalise well to other data without manually recalibrating the algorithm, which can take considerable time and present notable difficulties. On the other hand, a range of alternative methods have been proposed which are based on deep learning. A deep learning method can automatically learn the rules from a dataset, rectifying one of the main drawbacks of traditional analysis techniques. Additionally, extending an algorithm to new data typically requires simply extending the training set with the new samples, while most other training and model parameters do not need to be modified for the method to operate. There are numerous deep learning methods that have been proposed for OCT retinal segmentation with a few different techniques commonly employed including patch-based methods^10–14^ and semantic segmentation methods^14–26^, among others. Semantic segmentation methods, in particular, have demonstrated state-of-the-art performance in retinal and choroidal layer segmentation tasks of OCT images^14^.

The majority of semantic segmentation methods adopt an encoder-decoder deep neural network structure, most of which base their architectures on the U-Net^27^, improving over the original fully-convolutional network approach^28^. In the U-Net, skip connections improve gradient flow and allow the transfer of information between the down-sampling and up-sampling paths by connecting each pair of encoder-decoder layers. The U-Net takes an input OCT image and classifies each individual pixel in the image into one of several possible classes, thereby segmenting the image into the different regions. For OCT retinal layer segmentation, the classes correspond to the different tissue layers and other regions around the tissue layers of interest. The output of the U-Net is a pixel-level map providing precise segmentation of the regions and layers of interest.

There are several variants which aim to improve performance compared to the original (vanilla) U-Net architecture. Dense U-Net^29,30^ connects all convolution block outputs to the inputs of all subsequent blocks within each level which encourage feature reuse as well as aiding in preventing vanishing gradients. The Inception U-Net^31^ employs multiple convolutional kernel sizes in parallel paths at each level ensuring a greater level of robustness to images of different scales. The Attention U-Net^32^ employs an attention module within each skip connection, with these modules allowing for greater focus to be given to the more important spatial regions within an image and lesser focus on those of lower importance. The residual U-Net^33,34^ adopts residual learning in each of the layers by adding shortcut connections which aim to improve gradient flow and work under the theory that the residual mapping is easier to learn than the original one. The recurrent-residual (R2) U-Net^35^ combines the benefits of residual learning (see Residual U-Net) and recurrent connections, the latter of which allows for improved feature accumulation and parameter utilisation. The U-Net++^36^ uses a set of dense skip connections allowing for improved aggregation of features across different semantic scales. The squeeze + excite (SE) U-Net^37–39^ incorporates squeeze + excite blocks at the output of each convolutional block. These blocks allow for activation map reweighting of both spatial locations and channels based on the relative importance of each spatial location and channel. A summary of each architecture as well as references to their prior applications can be found in Table 1.

**Table 1:** Summary of the U-Net architectures compared in this study.

Variant	Original	Applications	Key features
Vanilla U-Net	^27^	^14,17,18,25^	Encoder-decoder, pixel-level semantic segmentation, skip connections
Dense U-Net	^29,30^	^20,40,41^	Encourage feature reuse, avoid vanishing gradients, connect each output to all subsequent inputs within the block
Inception U-Net	^31^	^42,43^	Multiple kernel sizes per layer, robust to images of different scales
Attention U-Net	^32^	^26,44–46^	Focus more on important features, focus less on unimportant ones
Residual U-Net	^33,34^	^14–16,47,14^	Shortcut connections, easier to optimise residual compared to original, improve gradient flow, avoid performance degradation in very deep networks
R2 U-Net	^35^	^48^	Benefits of residual learning + feature accumulation and improved parameter utilisation
U-Net + +	^36^	–	Dense skip connections, improved feature aggregation
SE U-Net	^37–39^	^14,15,24^	Spatial and channel recalibration, reweight spatial locations and feature maps (channels) based on importance

Previous studies for medical image segmentation (including OCT) have proposed a number of variants of the U-net architecture to improve performance. However, the effect of these U-Net architectural changes on OCT image segmentation performance is unclear, as an unbiased comparison across multiple OCT datasets has not been performed. Indeed, previous comparisons use different datasets to one another, compare using only a single dataset, compare only using a small subset of methods, and/or contain bias due to network architectures not being properly matched (e.g. using a different number of layers to one another).

In this study, a comparison is performed using eight significant U-Net variants on four different and varied OCT datasets to obtain an understanding of their effect on segmentation performance as well as trade-offs with respect to computational time (training and evaluation) and complexity. The four OCT datasets encompass data from a range of different populations, ocular pathologies, scanning parameters, instruments and segmentation tasks, to ensure that the comparison is not biased and limited to just a single dataset. The effect of the number of convolutional layers is also examined, by comparing two and three layers per block, as a simple complementary experiment alongside the more complex architectural changes. The overall goal of this study is to determine general conclusions for semantic OCT retinal layer segmentation using U-Net architectures which can be applied to any OCT dataset and result in significant time savings for future studies. This study also examines network architectures that have not previously been applied to this problem. To the best of our knowledge, the U-Net++, R2U-Net and Inception U-Net have not been previously applied to OCT retinal or choroidal segmentation and hence will be investigated in this study.

## Methods

### Data

Four datasets were used in this study, which aim to provide a wide range of image qualities and features. Thus, it will allow an improved understanding of the performance of the U-Net variants. For the relevant ethics information, please refer to the original studies using the citations below. All methods were performed in accordance with the relevant guidelines and regulations.

### Dataset 1: Healthy

**Figure 1:**
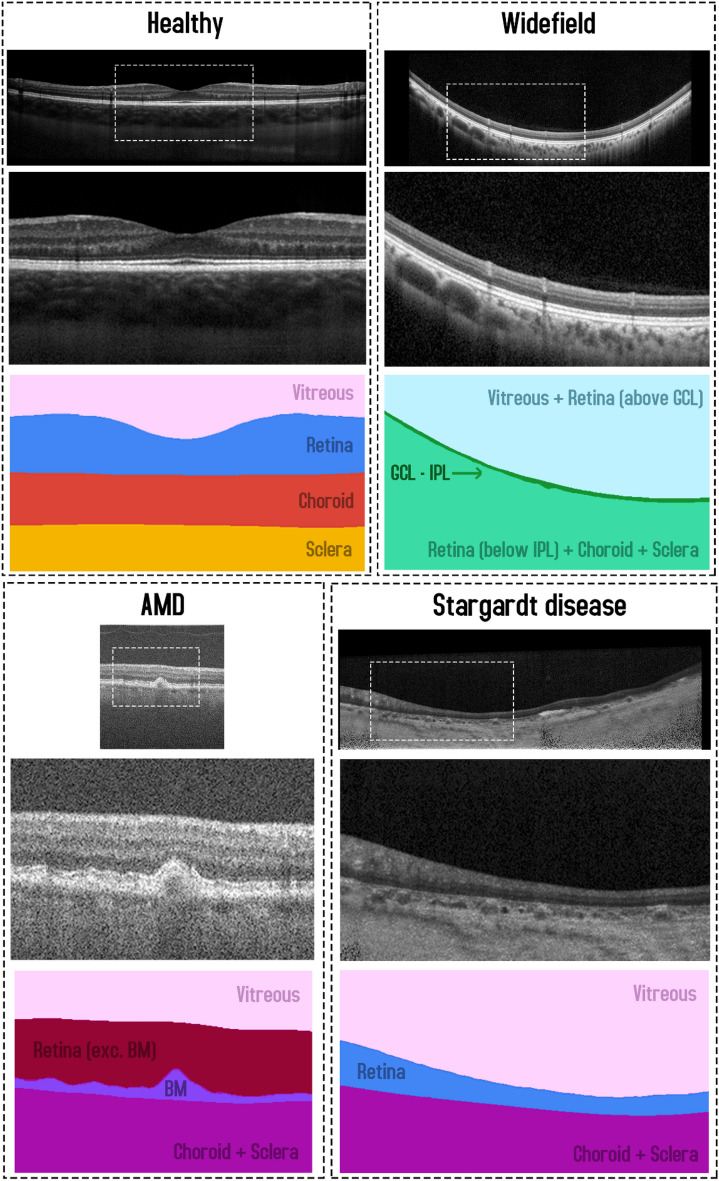
Example OCT image and corresponding semantic segmentation mask for each of the four datasets. Images are shown maintaining their aspect ratio. Each individual colour corresponds to a different region/class as labelled. White dashed box in the topmost corresponds to the zoomed region (second and third images) for each dataset.

This dataset, from a previous study^49^, consists of spectral domain OCT (SD-OCT) B-scans from 104 healthy children. Approval from the Queensland University of Technology human research ethics committee was obtained before commencement of the study, and written informed consent was provided by all participating children and their parents. All participants were treated in accordance with the tenets of the Declaration of Helsinki. Data was sourced across four separate visits over a period of approximately 18 months, however for the purposes of this study, we utilise data from the first visit only. For this visit, 6 cross-sectional scans were acquired, equally spaced in a radial pattern, and centred upon the fovea. Scans were acquired using the Heidelberg Spectralis SD-OCT instrument. For all scans, 30 frames were averaged using the inbuilt automated real time function (ART) to reduce noise while enhanced depth imaging (EDI) was used to improve the visibility of the choroidal tissue. Each scan measures 1536 pixels wide by 496 pixels deep (approximately 8.8 × 1.9 mm respectively in physical dimensions with a vertical scale of 3.9 µm per pixel and a horizontal scale of 5.7 µm per pixel). After data collection, all scans were annotated by an expert human observer with boundary annotations created for three tissue layer boundaries including: the inner boundary of the inner limiting membrane (ILM), the outer boundary of retinal pigment epithelium (RPE), and the choroid-sclera interface (CSI). For each scan, a semantic segmentation mask (same pixel dimensions) is constructed with four regions: (1) vitreous (all pixels between top of the image and the ILM), (2) retina (ILM to RPE), (3) choroid (RPE to CSI), and (4) sclera (CSI to bottom of the image). An example scan and corresponding segmentation mask is provided in Fig. 1. For the purposes of this study, the data was randomly divided into a training (40 participants, 240 scans total), validation (10 participants, 60 scans total) and testing set (49 participants, 294 scans total) with no participant’s data overlapping between sets.

### Dataset 2: Stargardt disease

This dataset, described in a prior study^15^, consists of SD-OCT scans of patients with varying stages of Stargardt disease. Approval to identify and use SD-OCT images from patients with genetically confirmed Stargardt disease for developing segmentation methods was obtained from the Human Ethics Office of Research Enterprise, The University of Western Australia (RA/4/1/8932 and RA/4/1/7916) and the Human Research Ethics Committee, Sir Charles Gairdner Hospital (2001-053). Scans were acquired from 18 participants, with 4 volumes (captured at different visits at different times) of ~ 50–60 B-scans from each. The scans measure 1536 pixels wide by 496 pixels high, with a vertical scale of 3.9 µm per pixel and a horizontal scale of 5.7 µm per pixel, this corresponds to an approximate physical area of size 8.8 × 1.9 mm (width × height). Scans were acquired using the Heidelberg Spectralis SD-OCT instrument with the ART algorithm used to enhance the definition of each B-scan by averaging nine OCT images. Scans were taken in high-resolution mode, unless it was determined necessary to use high-speed mode owing to poor fixation (any low-resolution scan was resized to match the resolution of the dataset). EDI was not employed. Contrast enhancement is utilised^15,50^ in an effort to improve layer visibility and subsequently improve segmentation performance. After acquisition, each scan was annotated by an expert human observer, with annotations provided for two layer boundaries including: the inner boundary of the inner limiting membrane (ILM), and the outer boundary of the retinal pigment epithelium (RPE). In some patients with Stargardt disease, the RPE and the outer retinal layers are lost in the central region of the retina. In such cases, the remaining Bruch’s membrane (BM) that separates the residual inner retina from the choroid is marked as the outer boundary. For each scan, a semantic segmentation mask is constructed (same pixel dimensions) with three regions: (1) vitreous (all pixels from top of image to the ILM), (2) retina (from ILM to RPE/BM) and (3) choroid/sclera (RPE/BM to bottom of the image). An example scan and corresponding segmentation mask is provided in Fig. 1. Each participant is categorised into one of two categories based on retinal volume (low or high). This is calculated based on the total macular retinal volume based on the boundary annotations such that there is an even number of participants in each category. For this study, the data is divided into training (10 participants, 2426 scans), validation (2 participants, 486 scans), and testing (6 participants, 1370 scans) sets ensuring that there is an even split of low and high volume participants in each set.

### Dataset 3: Age-related macular degeneration

This dataset consists of OCT scans of patients exhibiting age-related macular degeneration (AMD)^51^. All scans were acquired using the Bioptigen SD-OCT with data sourced from four different sites with scanning resolutions varying slightly between the sites^52^. No image averaging is employed. A total of 269 participants are utilised, each with a single volume of 100 scans. However, only a subset of the scans is used. A scan is used only if it contains at least 512 pixels (approximately half the width) of available boundary annotations, otherwise it is discarded. Each scan is then cropped to a central 512 pixels horizontally with each scan also measuring 512 pixels in height (no cropping). Each scan is supplied with boundary annotations for three layer boundaries including the inner boundary of the ILM, the outer boundary of the retinal pigment epithelium drusen complex (RPEDC) and the outer boundary of BM. For each scan, a semantic segmentation mask is constructed (same pixel dimensions) with four regions including: (1) vitreous (all pixels from the top of the image to the ILM), (2) retina (ILM to RPEDC), (3) Bruch’s membrane (RPEDC to BM), and (4) choroid/sclera (BM to bottom of the image). An example scan and corresponding segmentation mask is provided in Fig. 1. A total of 163 participants (4439 scans) are used for training, 54 participants (1481 scans) for validation, and 52 participants (1421 scans) for testing with all participants assigned randomly with no overlap or duplication.

### Dataset 4: Widefield

This dataset, which has been described in detail elsewhere^53^, included 12 healthy participants that had widefield OCT volume scans acquired using a widefield objective lens while maintaining central fixation. All participants provided written consent as per protocols approved by the University of New South Wales Australia Human Research Ethics Advisory panel, and the study adhered to the tenets of the Declaration of Helsinki. Scans were acquired using the Heidelberg Spectralis where the applied scanning protocol acquired 109 B-scans spaced 120 μm apart, spanning a total area of 55° horizontally and 45° vertically (15.84 mm wide and 12.96 mm high), and ART was set to 16 to reduce noise. OCT B-scans were manually segmented to extract two boundaries of interest: the inner boundary of the ganglion cell-inner layer (GCL), and the outer boundary of the inner plexiform layer (IPL). The thickness data between these two boundaries (ganglion cell-inner plexiform layer thickness) can inform the detection and screening of a number of retinal diseases such as glaucoma^54^, central retinal vein occlusion^55^ and diabetic macular edema^56^. After resizing and cropping to the region of interest, the scans measure 1392 pixels wide by 496 pixels high. For each scan, a semantic segmentation mask is constructed (same pixel dimensions) with three regions: (1) all pixels from the top of the image to the GCL, (2) all pixels between GCL and IPL, and (3) all pixels between the IPL and the bottom of the image. An example scan and corresponding segmentation mask is provided in Fig. 1. Scans from a total of 12 participants (~ 109 scans each, with one scan discarded from a single participant) are utilised with 6 participants assigned for training (654 images total), 3 for validation (326 images total) and 3 for testing (327 images total). There is no overlap of participants between the three sets.

### Training and architecture parameters

**Figure 2:**
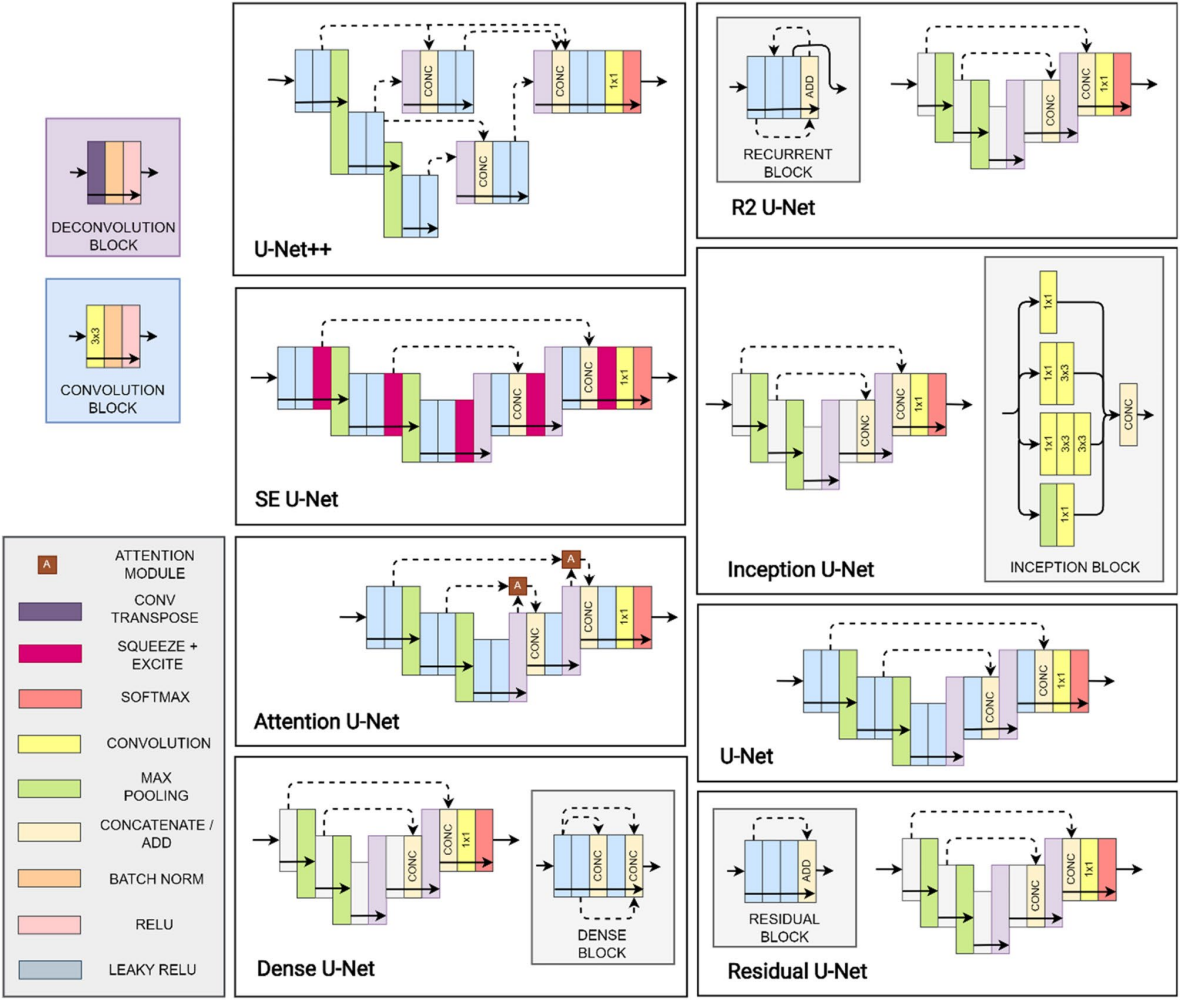
Summary of the U-Net based architectures compared in this study. Each architecture is depicted with two pooling layers for simplicity but each uses four pooling layers in our experiments. Arrows show direction of information flow. Coloured blocks represent particular layers as per the legend. Note: The R2, Residual and Dense variants are depicted with three convolutional layers per block (for improved visualisation) while the others are all depicted with two (except for the Inception variant where the number of layers does not apply).

All networks are trained to perform semantic segmentation of the OCT scans. Using a 1 × 1 convolution layer (1 × 1 strides, filter count corresponding to number of regions) followed by a softmax activation, the output of each network consists of class probabilities corresponding to each pixel in the original input OCT image. For the Stargardt disease and widefield datasets, the probabilities represent the classification of each pixel into one of three areas/regions of the OCT images, while there are four such areas for the healthy and AMD datasets as described in the Data section. For each of the eight variants of the U-net architecture that are considered, we build off previous code implementations as follows: (1) baseline^57^, (2) dense^58^, (3) attention^59^, (4) squeeze and excite (SE)^60^, (5) residual^61^, (6) recurrent-residual (R2)^62^, (7) U-Net++^63^, and (8) Inception^64^. For the comparison in this study, all networks utilise max pooling layers (2 × 2) for down-sampling and transposed convolutions for up-sampling (2 × 2 kernel and strides). Each network consists of four pooling/up-sampling layers with 16 filters for all convolutions in the first layer which is doubled after each pooling and subsequently halved at each up-sampling layer. Each convolution uses Glorot uniform^65^ kernel initialisation, and each layer, with the exception of the output layer, is followed by batch normalisation^66^ and a rectified linear unit activation. We compare two variants for each architecture by considering two and three convolutions per layer. A visual summary of each architecture is given in Fig. 2. For the residual variant, the ‘ReLU before addition’ block variant is selected while two recurrences are performed for the R2 variant. For the SE variant, we employ the scSE module (concurrent cSE and sSE) with default ratio of 2 for the cSE component. The U-Net++ architecture employs the same filters within the encoder and decoder (16 filters first layer, subsequently doubled in each layer) as the other architectures. However, all the intermediate layers (between the encoder and decoder) use 16 filters each, for computational reasons.

For training, the Adam optimizer^67^ is employed with default parameters (except for the learning rate which is set at 0.0001). A batch size of two is used and all networks are trained for 200 epochs, minimising cross-entropy as the training objective. For model selection, we use the epoch with the best Dice coefficient on the validation set. The Dice coefficient is a measure of similarity between two samples (in this case, the predicted segmentation map and ground truth map) and is defined as:

1 $$Dice=\frac{2TP}{2TP+FP+FN}$$

where TP: number of true positives, FP: number of false positives, and FN: number of false negatives.

For regularisation during training, all training samples are shuffled randomly at the beginning of each epoch. No augmentation is employed for any experiments for simplicity. The comparison between architectures is performed by comparing the Dice coefficient on the testing set using the model selected from the best epoch. To perform a fair comparison between the network architectures, it is necessary to run each experiment several times to ensure that there is no bias as a result of random weight initialisation and sample shuffling. Hence, all experiments are performed three times in this study. All experiments are undertaken using Tensorflow 2.3.1 in Python 3.7.4 using an NVIDIA M40 graphics processing unit (GPU).

To statistically compare the segmentation outcomes (Dice coefficient) from the different architectures, a one-way repeated measures analysis of variance (ANOVA) was run for each of the different datasets for both the 2 layer and 3 layer variants separately, examining the within-subject effect of network architecture. Bonferroni-adjusted pairwise comparisons were conducted to compare between specific network architectures. An additional ANOVA was conducted to compare between the segmentation outcomes of the 2 layer and 3 layer variants.

## Results

**Figure 3:**
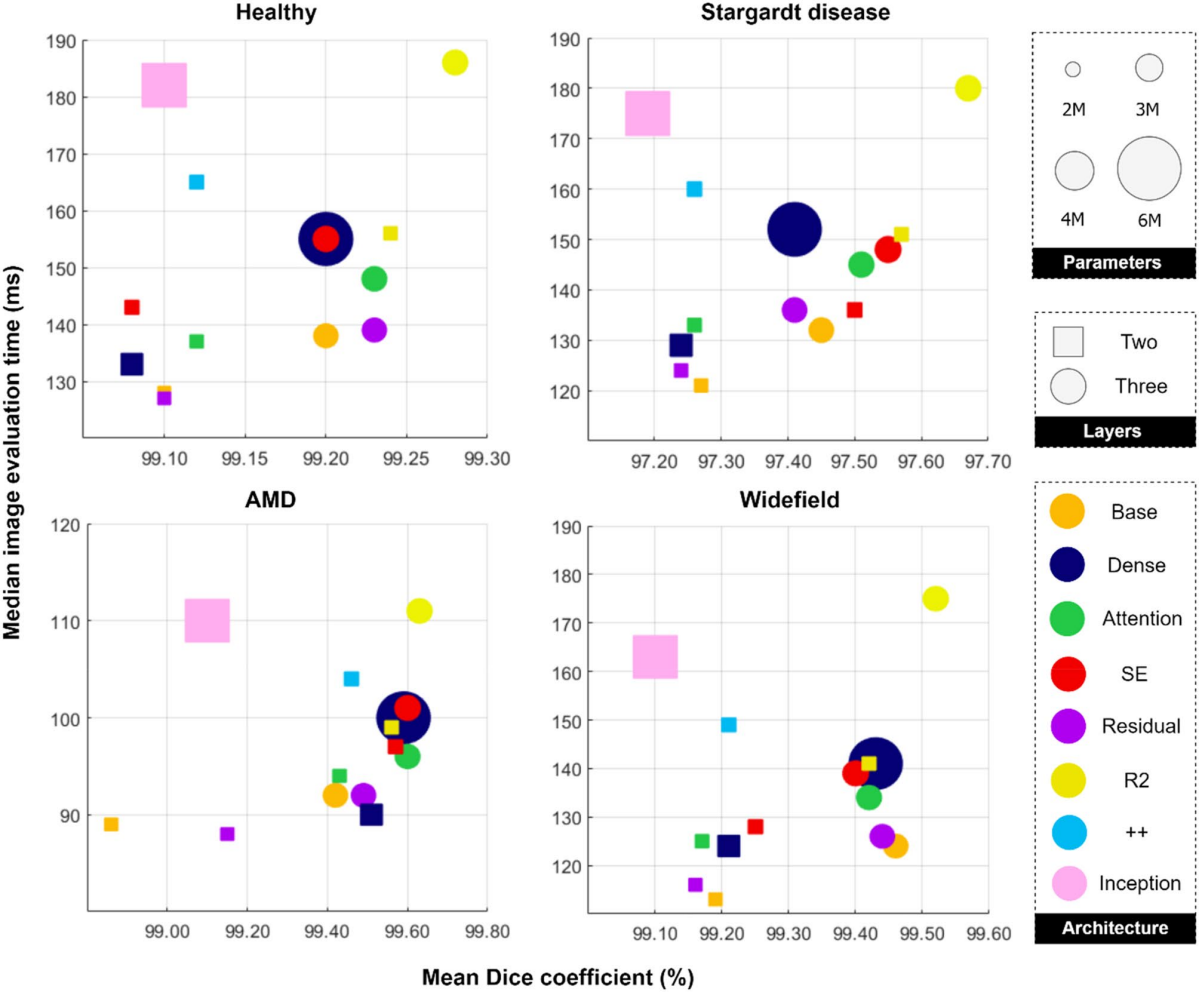
Segmentation accuracy (horizontal axis) vs. evaluation speed (vertical axis) vs. network parameters (size of symbol) for each of the datasets for two and three layers (square and circle symbols respectively) for each architecture tested. Note: the axis scales on each subplot differ to support optimal visualisation.

**Figure 4:**
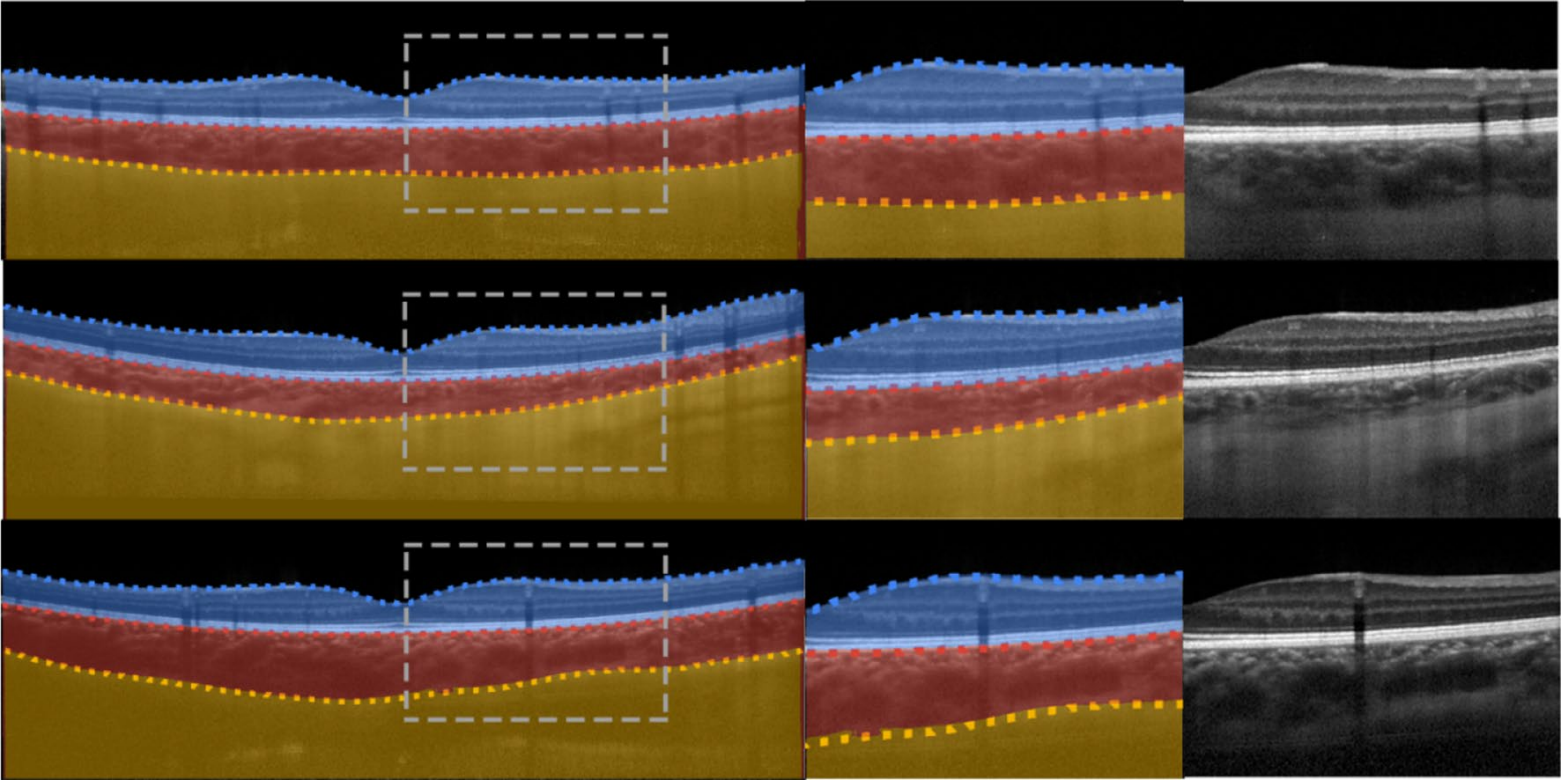
Example segmentation outputs from the R2 U-Net (3 layer variant) for the healthy dataset overlaid on the images. Left: full image with grey dashed rectangle corresponding to the zoomed area of the two rightmost images. Blue region: retina, red region: choroid, yellow region: sclera. Dotted lines correspond to the boundary position ground truths.

**Figure 5:**
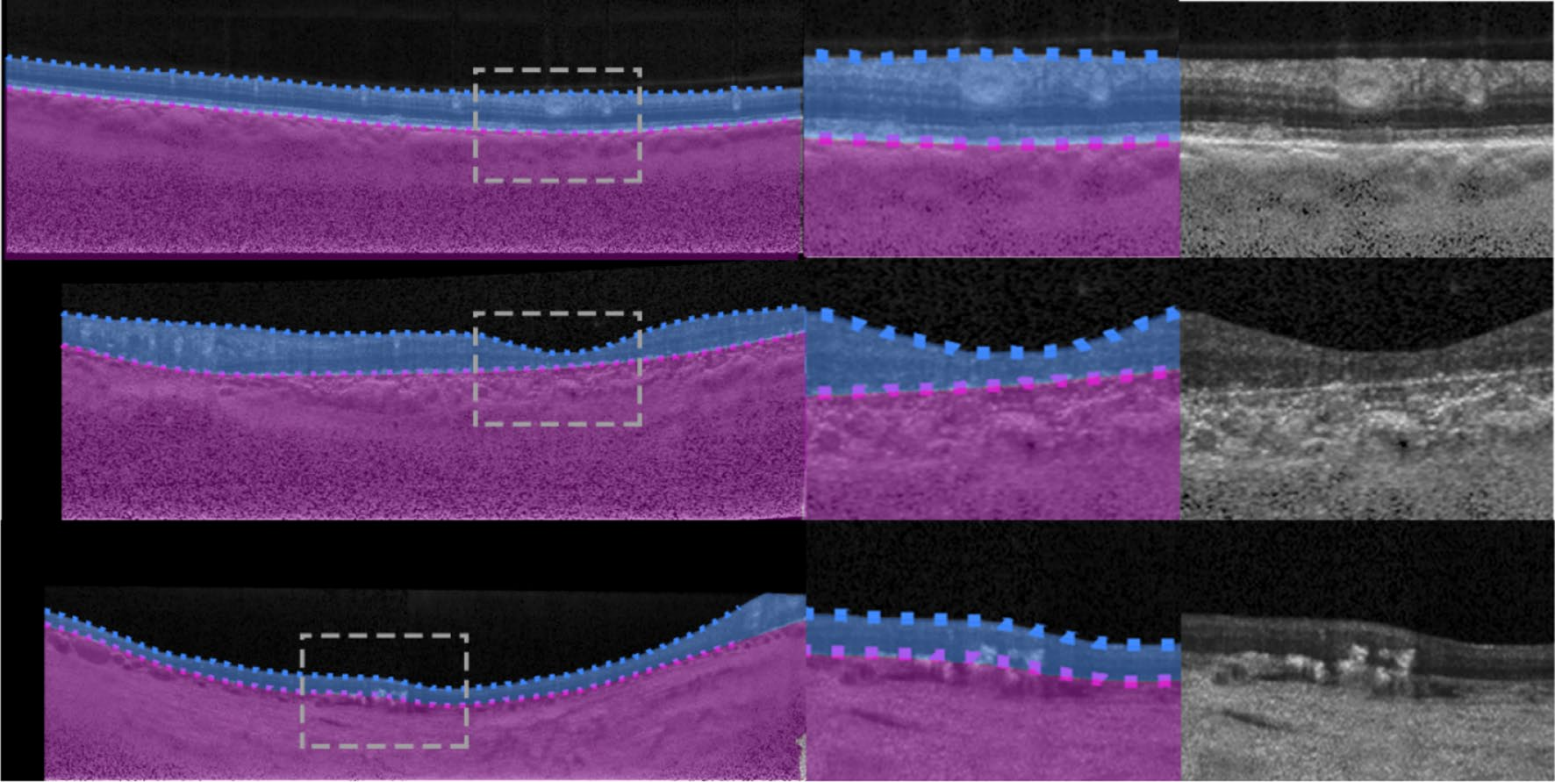
Example segmentation outputs from the R2 U-Net (3 layer variant) for the Stargardt disease dataset overlaid on the images. Left: full image with grey dashed rectangle corresponding to the zoomed area of the two rightmost images. Blue region: retina, purple region: choroid + sclera. Dotted lines correspond to the boundary position ground truths.

**Figure 6:**
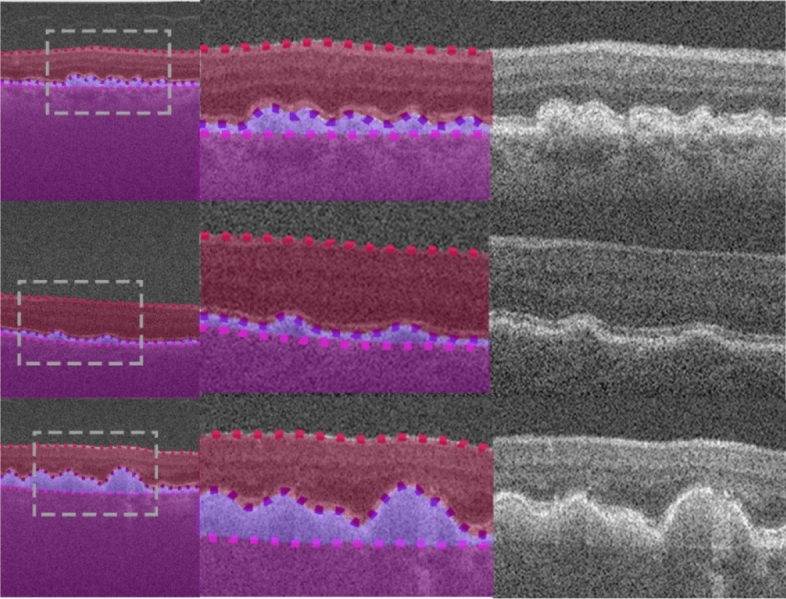
Example segmentation outputs from the R2 U-Net (3 layer variant) for the AMD dataset overlaid on the images. Left: full image with grey dashed rectangle corresponding to the zoomed area of the two rightmost images. Red region: retina (excluding BM), light purple region: BM, dark purple region: choroid + sclera. Dotted lines correspond to the boundary position ground truths.

**Figure 7:**
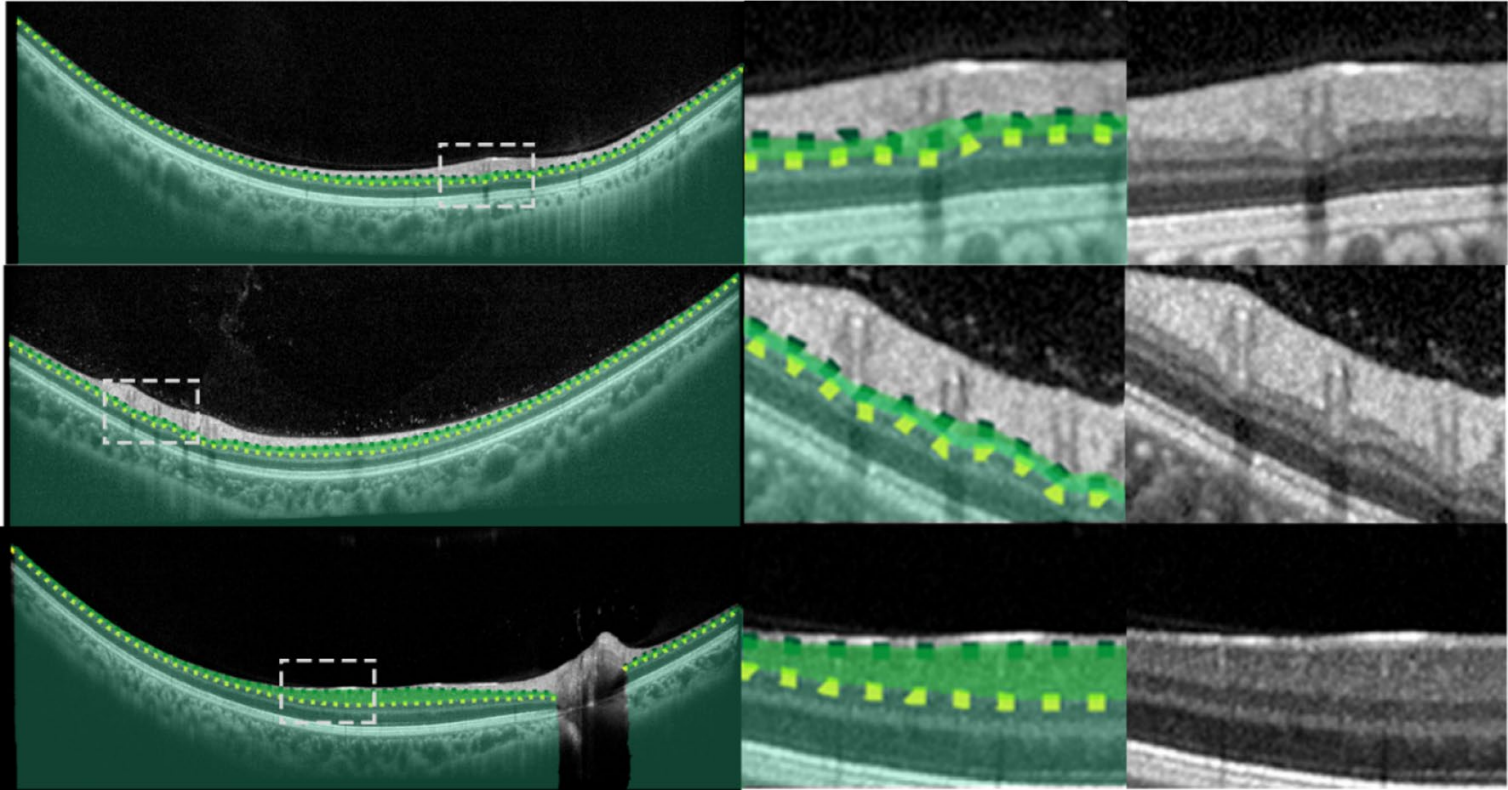
Example segmentation outputs from the R2 U-Net (3 layer variant) for the widefield dataset overlaid on the images. Left: full image with grey dashed rectangle corresponding to the zoomed area of the two rightmost images. Bright green region: GCL-IPL, dark green region: retina (below IPL) + choroid + sclera. Dotted lines correspond to the boundary position ground truths.

Tables 2, 3, 4 and 5 provide a summary of the main results for the healthy dataset, Stargardt disease dataset, AMD dataset and widefield dataset, respectively. The accuracy represents the mean (and standard deviation) overall Dice coefficient as the main performance metric, while the times (epoch training and evaluation time) together with the number of parameters allow for a comparison of the computational complexity of the networks. Figure 3 provides a visual summary of accuracy vs. evaluation time vs. network complexity with a subplot for each of the four datasets. It can be noted that the general clustering of the two-layer variants (squares) and three-layer variants (circles) indicates that most architectures exhibit comparable segmentation accuracy (x-axis) with the notable exception being the R2 variant (in yellow), which showed a marginal performance improvement. Additionally, the general difference between these two clusters (squares and circles) indicates that the three-layer variant (circles) consistently outperforms the two-layer variant (squares), but only by a small amount. While the R2 variant (yellow) is the most accurate, it is also the slowest with respect to evaluation time (y-axis) and therefore lies in the top right corner of the subplots. However, this is not a general trend with the Inception variant, for instance, lying in the top left corner of the subplots (slow but with relatively low accuracy compared to the other variants). Observing the subplots, there does not appear to be any clear trends with respect to the number of network parameters (relative sizes of the circles and squares). Figures 4, 5, 6, and 7 give some example segmentation outputs overlaid with transparency on the original OCT scans and comparisons to ground truth segmentation, demonstrating the robustness and accuracy of the segmentations for each of the four datasets (healthy, Stargardt disease, AMD and widefield) respectively using the R2 U-Net (3 layer variant).

**Table 2:** Comparison of results for the healthy dataset.

Network architecture	Conv/layer	Accuracy (SD) [%]	Mean epoch train time (SD) [s]	Median image evaluation time [msec]	Parameters [$\times {10}^{6}$]
Vanilla U-Net	2	99.10 (**0.01**)	**41.46** (0.60)	128	**1.95**
	3	99.20 (0.05)	54.33 (0.34)	138	2.93
Dense U-Net	2	99.08 (0.02)	46.36 (0.14)	133	2.71
	3	99.20 (0.05)	72.88 (**0.02**)	155	5.44
Attention U-Net	2	99.12 (0.05)	53.00 (0.85)	137	1.99
	3	99.23 (**0.01**)	66.65 (0.52)	148	2.98
SE U-Net	2	99.08 (0.02)	57.92 (0.95)	143	2.06
	3	99.20 (0.06)	70.88 (0.27)	155	3.04
Residual U-Net	2	99.10 (0.04)	42.61 (0.07)	**127**	**1.95**
	3	99.23 (0.02)	55.50 (0.12)	139	2.93
R2 U-Net	2	99.24 (0.02)	65.01 (1.04)	156	2.00
	3	**99.28** (**0.01**)	103.59 (0.23)	186	3.00
U-Net + +		99.12 (0.02)	93.23 (1.38)	165	2.05
Inception U-Net		99.10 (0.04)	108.31 (0.04)	182	4.62

(SD: standard deviation, s: seconds, SE: squeeze + excite, R2: recurrent-residual). The best and poorest results for each are annotated in bold and underline text respectively.

**Table 3:** Comparison of results for the Stargardt disease dataset.

Network architecture	Conv/layer	Accuracy (SD) [%]	Mean epoch train time (SD) [s]	Median image evaluation time [msec]	Parameters [$\times {10}^{6}$]
Vanilla U-Net	2	97.27 (0.07)	**366.89** (1.09)	**121**	**1.95**
	3	97.45 (0.03)	509.44 (1.11)	132	2.93
Dense U-Net	2	97.24 (0.02)	422.02 (**0.61**)	129	2.71
	3	97.41 (0.07)	696.60 (5.07)	152	5.44
Attention U-Net	2	97.26 (**0.01**)	505.11 (3.91)	133	1.99
	3	97.51 (0.03)	631.13 (2.50)	145	2.98
SE U-Net	2	97.50 (0.07)	541.35 (5.45)	136	2.06
	3	97.55 (0.06)	674.84 (2.15)	148	3.04
Residual U-Net	2	97.24 (0.04)	388.44 (4.56)	124	**1.95**
	3	97.41 (0.02)	524.82 (3.80)	136	2.93
R2 U-Net	2	97.57 (0.06)	605.62 (0.76)	151	2.00
	3	**97.67** (0.05)	1004.66 (3.46)	180	3.00
U-Net + +		97.26 (0.05)	885.71 (4.98)	160	2.05
Inception U-Net		97.19 (0.05)	1047.61 (3.95)	175	4.62

(SD: standard deviation, s: seconds, SE: squeeze + excite, R2: recurrent-residual). The best and poorest results for each are annotated in bold and underline text respectively.

**Table 4:** Comparison of results for the AMD dataset.

Network architecture	Conv/layer	Accuracy (SD) [%]	Mean epoch train time (SD) [s]	Median image evaluation time [msec]	Parameters [$\times {10}^{6}$]
Vanilla U-Net	2	98.86 (0.63)	**252.74** (0.88)	89	**1.95**
	3	99.42 (0.29)	339.31 (**0.11**)	92	2.93
Dense U-Net	2	99.51 (0.07)	291.18 (2.37)	90	2.71
	3	99.59 (0.03)	463.66 (1.71)	100	5.44
Attention U-Net	2	99.43 (0.21)	341.10 (5.71)	94	1.99
	3	99.60 (0.02)	427.15 (7.47)	96	2.98
SE U-Net	2	99.57 (0.02)	388.54 (4.60)	97	2.06
	3	99.60 (0.02)	468.74 (4.84)	101	3.04
Residual U-Net	2	99.15 (0.54)	268.15 (4.26)	**88**	**1.95**
	3	99.49 (0.13)	352.40 (3.42)	92	2.93
R2 U-Net	2	99.56 (**0.01**)	409.71 (1.43)	99	2.00
	3	**99.63** (0.02)	671.08 (3.52)	111	3.00
U-Net + +		99.46 (0.05)	588.80 (2.19)	104	2.05
Inception U-Net		99.10 (0.33)	686.44 (2.89)	110	4.62

SD: standard deviation, s: seconds, SE: squeeze + excite, R2: recurrent-residual). The best and poorest results for each are annotated in bold and underline text respectively.

**Table 5:** Comparison of results for the widefield dataset.

Network architecture	Conv/layer	Accuracy (SD) [%]	Mean epoch train time (SD) [sec]	Median image evaluation time [msec]	Parameters [$\times {10}^{6}$]
Vanilla U-Net	2	99.19 (0.03)	**102.34** (1.58)	**113**	**1.95**
	3	99.46 (0.02)	138.03 (1.98)	124	2.93
Dense U-Net	2	99.21 (0.04)	122.12 (0.27)	124	2.71
	3	99.43 (0.03)	192.76 (0.97)	141	5.44
Attention U-Net	2	99.17 (0.06)	136.72 (**0.04**)	125	1.99
	3	99.42 (0.06)	171.68 (3.00)	134	2.98
SE U-Net	2	99.25 (0.02)	146.67 (0.46)	128	2.06
	3	99.40 (0.06)	181.86 (1.79)	139	3.04
Residual U-Net	2	99.16 (0.04)	108.19 (1.31)	116	**1.95**
	3	99.44 (0.02)	140.31 (0.43)	126	2.93
R2 U-Net	2	99.42 (0.04)	165.19 (1.38)	141	2.00
	3	**99.52** (0.07)	267.28 (0.63)	175	3.00
U-Net + +		99.21 (**0.01**)	234.88 (1.14)	149	2.05
Inception U-Net		99.10 (0.13)	277.68 (1.35)	163	4.62

(SD: standard deviation, s: seconds, SE: squeeze + excite, R2: recurrent-residual). The best and poorest results for each are annotated in bold and underline text respectively.

The repeated measures ANOVA revealed a significant effect of network architecture for both the healthy and Stargardt disease datasets (for both the 2 and 3 layer variants). Although the magnitude of differences were small, the R2 architecture was found to be statistically significantly more accurate compared to some of the other architectures including the vanilla, attention, SE and residual U-Net architectures (all *p* < 0.05). There were also statistically significant differences for each architecture comparing the two and three layer variants with the three-layer variant being slightly but statistically significantly more accurate on both the healthy and Stargardt disease datasets (*p* < 0.05). Additionally, it was found that using three layers (compared to two layers) was significantly more accurate overall (not taking architecture into account) (*p* < 0.05) on the healthy, Stargardt disease and widefield datasets. On the other hand, there were no statistically significant differences between architectures for the AMD and widefield datasets (all *p* > 0.05). The lack of significance of the results on these datasets is likely related to the more variable performance on the AMD dataset and the small number of participants in the widefield dataset.

## Discussion

In this study, eight different U-Net variants were compared for their segmentation performance on four different OCT image datasets encompassing a range of ocular pathologies and scanning parameters. Each architecture was also compared using both two and three convolutional blocks per layer. The results suggest that there is largely comparable performance (measured using Dice coefficient) for all of the architectures on the individual datasets. Indeed, despite the increased complexity and running time of a number of the U-Net variants, their performance was not notably greater than the baseline vanilla U-Net, which already exhibits excellent performance for OCT retinal layer segmentation.

The eight U-Net architectures were similar in terms of accuracy, and the performance of each improved slightly on all four datasets by using an additional convolutional layer per block (three instead of two), a comparatively simple architectural change. However, this was again at the cost of increased complexity and running time as a result of the extra parameters introduced by the additional layers. Despite the resultant performance improvements for each individual network, the comparison between each of them remains relatively unchanged. That is, their relative performance to one another is comparable as it is with two convolutional layers. The findings further highlight the importance of comparisons between different network architectures being performed carefully (e.g. using the same number of layers). For instance, a vanilla U-Net and a Residual U-Net are often fitted (by default) with two and three convolutional layers per block, respectively, which will potentially bias the performance of the Residual U-Net. In this specific scenario, the additional layer is the cause of improvement as opposed to the addition of residual connections. It may be possible for additional layers (e.g. 4 or more) to be added to further improve performance, however, this is at the cost of drastically increased model complexity as well as slower inference and training time. At a certain point, this increased complexity will also result in the inability of the computing hardware to handle the model size (i.e. insufficient VRAM). Additionally, we hypothesise that diminishing returns are likely with respect to performance (given the excellent performance with 3 layers) making the trade-off between performance and complexity even less favourable with higher numbers of convolutional layers.

While segmentation accuracy (Dice coefficient) is a critical metric, it is not the only one that should be considered when comparing these architectures. Indeed, training time, evaluation speed, and model complexity are some other vital factors to consider from the perspective of real-world training and model deployment both in research and clinical practice. Small performance improvements may not be worthwhile in certain applications if this is obtained at the cost of significantly increased complexity and reduced speed. In this case, while the vanilla U-Net is the fastest to train, one of the fastest to evaluate, and possesses the fewest parameters (lowest complexity), its performance is still comparable and competitive with the majority of the other architectures across all datasets. On the other hand, the R2 U-Net performs slightly better and contains only slightly more parameters but is significantly slower to train and evaluate as a result of the recurrences where layers are reused, requiring additional computation at each step. In fact, it is the slowest to evaluate of any of the architectures highlighting a key trade-off when selecting this architecture. Overall, the Inception U-Net does not perform favourably in any metric possessing significantly more parameters than most of the other networks, being the slowest to train and one of the slowest to evaluate, and yielding comparable performance on all of the datasets. Similarly, although the Dense U-Net uses more parameters than most of the other networks (as it takes in more feature maps in the subsequent dense block layers), its performance is only comparable across all four datasets. As expected, there is a high correlation between training and evaluation time but there are a few minor exceptions. Although the Inception U-Net is the slowest to train, the R2 U-Net (3 layer variant) is the slowest to evaluate across all four datasets. This is likely due to differences in the type of operations that are performed in the forward pass (used in evaluation) vs. in backpropagation (not used in evaluation). Based on these findings there is clear a trade-off between memory, training time, evaluation speed, and performance. However, the improvements in performance are small across all tested datasets and models, suggesting that simpler, faster models are the optimal choices for OCT retinal layer segmentation.

The general level of performance between the datasets varies. However, this is expected given the higher difficulty and subjectivity associated with images exhibiting pathology and with poorer image quality (i.e. varying noise levels). The comparison between architectures on some datasets told a slightly different story in some cases. For instance, we observe a small benefit to using the squeeze + excitation U-Net on the Stargardt disease dataset, similar to a previous study^15^. It appears that the recalibration of feature maps is beneficial in the presence of highly pathological data and where the overall segmentation accuracies (Dice coefficients) are notably lower than the other datasets. However, the overall level of improvement is small. In general, the performance between the three runs for each experiment was largely consistent, with standard deviations of less than 0.1% on three of the four datasets with respect to the Dice coefficient. However, the performance on the AMD dataset shows greater variability, particularly for the case of two convolutional layers on some of the architectures. Indeed, the vanilla, residual, attention and inception variants all exhibited standard deviations > 0.2% when outfitted with two convolutional layers. Adding a third convolutional layer showed a marked reduction in variability on all architectures (except Inception, where the number of layers does not apply). We hypothesise that this variability is caused by the greater noise present in the AMD dataset, with this problem rectified significantly with the increased network learning capacity (i.e. by adding an extra layer). We note that there are also differences in the training and inference speeds for each of the different datasets which are associated with differing quantities of data and different sized images respectively. However, these differences are irrelevant for an architectural comparison which examines a single dataset at a time, and has no effect on the overall conclusions of this study.

In this study, four datasets were employed that are highly representative of real world OCT datasets that may be encountered in practice. These are also similar to those used in other studies involving deep learning based OCT segmentation methods^11,12,14,15^. Some of these datasets contain only a few participants, indicative of the common difficulty of sourcing large numbers of participants due to privacy and confidentiality reasons as well as a lack of readily available patients exhibiting less common ocular pathologies. However, a significant number of scans from each of these patients are used, to support invariance of the model to patient agnostic image features and artefacts, such as speckle noise. Despite potential issues surrounding a low number of participants and model bias, the performance of all models is excellent across all datasets suggesting that this does not degrade the performance. Additionally, the findings from the architectural comparison on each dataset are largely comparable, demonstrating that the lower quantity of data on some datasets does not appear to be biasing the results or invalidating the findings of this study. This also further supports the previously demonstrated fact that the U-Net can be trained well even with a relatively small number of images^27^.

Here, there was an exclusive focus on an architectural comparison between U-Net variants for OCT semantic segmentation of retinal layers. We stress that there are other OCT segmentation methods that have not been considered here. These specific methods are not considered as they incorporate other changes and modifications beyond the architecture (e.g. loss function, class weighting, additional post-processing etc.) which are outside the scope of this study. For instance, another study^68^ has performed a comparison between traditional (Dice and logistic) and adversarial (GAN) losses. Future studies should examine other such parameters and perform similar comparisons. There are also numerous other configurations of U-Net architectures (e.g. different residual block configurations, number of recurrences) and other semantic segmentation architectures (e.g. DeepLabv3+^69^, SegNet^70^) that have not been tested here to retain a manageable scope for this study. An interesting direction for future work could be to examine an ensemble approach using different U-Net architectures for each ensemble member. However, given the largely comparable performance between the tested architectures in this study, we speculate that any performance benefits may be minor.

## Conclusion

In this study, we have performed a comprehensive, unbiased comparison of U-Net architecture variants for the application of semantic segmentation in retinal OCT images across several datasets. All tested U-Net architectures provide excellent performance for OCT retinal layer segmentation, and the results suggest that there is little notable difference in performance between them when evaluated using the Dice metric. There are expected trade-offs between performance, speed and complexity that are important to consider depending on the particular clinical and research application as well as constraints on time and available hardware. The findings of this study also highlight the importance of careful and unbiased comparisons of deep learning methods and correctly matching network architectures to obtain a true understanding on the impact of network architectural changes. Overall, the significantly increased complexity and reduced speed of the U-Net variants with only marginal performance gains suggest that the baseline vanilla U-Net is an optimal choice for OCT retinal layer segmentation in practice. State-of-the-art models do not appear to provide a clear benefit for this application while increasing the number of layers in each model resulted in small performance gains but, again, with a trade-off with respect to complexity and speed. A significant time investment in any study involving deep learning is that of model comparison and optimal selection. The findings in this study can help to provide a solid, well-informed starting point, alleviating the time and cost burden of experimental comparison in future studies while also guiding model selection towards simpler and faster models. The findings here are largely consistent across several varied OCT datasets with differing pathologies, instruments, scanning parameters and segmentation tasks. Therefore, these can be generalised and are likely transferable to a wide range of other OCT datasets further highlighting the benefit of this study for future work with U-Net based models for OCT retinal layer segmentation which are commonly employed for this application.

## Data Availability

The healthy, Stargardt disease and widefield datasets analysed during the current study are currently not publicly available. However, the algorithms and code used throughout this study are publicly and readily available at https://github.com/jakugel/unet-variants.
